# Protocol for the Pregnancy During the COVID-19 Pandemic (PdP) Study: A Longitudinal Cohort Study of Mental Health Among Pregnant Canadians During the COVID-19 Pandemic and Developmental Outcomes in Their Children

**DOI:** 10.2196/25407

**Published:** 2021-04-28

**Authors:** Gerald F Giesbrecht, Mercedes Bagshawe, Melinda van Sloten, Anna L MacKinnon, Ashley Dhillon, Marcel van de Wouw, Elnaz Vaghef-Mehrabany, Laura Rojas, Danielle Cattani, Catherine Lebel, Lianne Tomfohr-Madsen

**Affiliations:** 1 Department of Pediatrics University of Calgary Calgary, AB Canada; 2 Alberta Children's Hospital Research Institute Calgary, AB Canada; 3 Department of Psychology University of Calgary Calgary, AB Canada; 4 Department of Community Health Sciences University of Calgary Calgary, AB Canada; 5 Department of Radiology University of Calgary Calgary, AB Canada

**Keywords:** pregnancy, anxiety, depression, stress, social support, resilience, COVID-19, infant development, pandemic

## Abstract

**Background:**

The COVID-19 pandemic and countermeasures implemented by governments around the world have led to dramatically increased symptoms of depression and anxiety. Pregnant individuals may be particularly vulnerable to the negative psychological effects of COVID-19 public health measures because they represent a demographic that is most affected by disasters and because pregnancy itself entails significant life changes that require major psychosocial and emotional adjustments.

**Objective:**

The PdP study was designed to investigate the associations among exposure to objective hardship caused by the pandemic, perceived stress and psychological distress in pregnant individuals, and developmental outcomes in their offspring.

**Methods:**

The PdP study comprises a prospective longitudinal cohort of individuals who were pregnant at enrollment, with repeated follow-ups during pregnancy and the postpartum period. Participants were eligible if they were pregnant, ≥17 years old, at ≤35 weeks of gestation at study enrollment, living in Canada, and able to read and write in English or French. At enrollment, participants completed an initial survey that assessed demographic and socioeconomic characteristics, previous pregnancies and births, prepregnancy health, health conditions during pregnancy, medications, psychological distress, social support, and hardships experienced because of the COVID-19 pandemic (eg, lost employment or a loved one dying). For the first three months following the initial survey, participants received a monthly email link to complete a follow-up survey that asked about their experiences since the previous survey. After three months, follow-up surveys were sent every other month to reduce participant burden. For each of these surveys, participants were first asked if they were still pregnant and then routed either to the next prenatal survey or to the delivery survey. In the postpartum period, surveys were sent at 3, 6, and 12 months of infant age to assess maternal stress, psychological distress, and infant development.

**Results:**

Participant recruitment via social media (Facebook and Instagram) began on April 5, 2020, and is ongoing. As of April 2021, more than 11,000 individuals have started the initial survey. Follow-up data collection is ongoing.

**Conclusions:**

This longitudinal investigation seeks to elucidate the associations among hardships, maternal psychological distress, child development during the COVID-19 pandemic, and risk and resilience factors that amplify or ameliorate these associations. The findings of this study are intended to generate knowledge about the psychological consequences of pandemics on pregnant individuals and point toward prevention and intervention targets.

**International Registered Report Identifier (IRRID):**

DERR1-10.2196/25407

## Introduction

### Background

**Figure 1:**
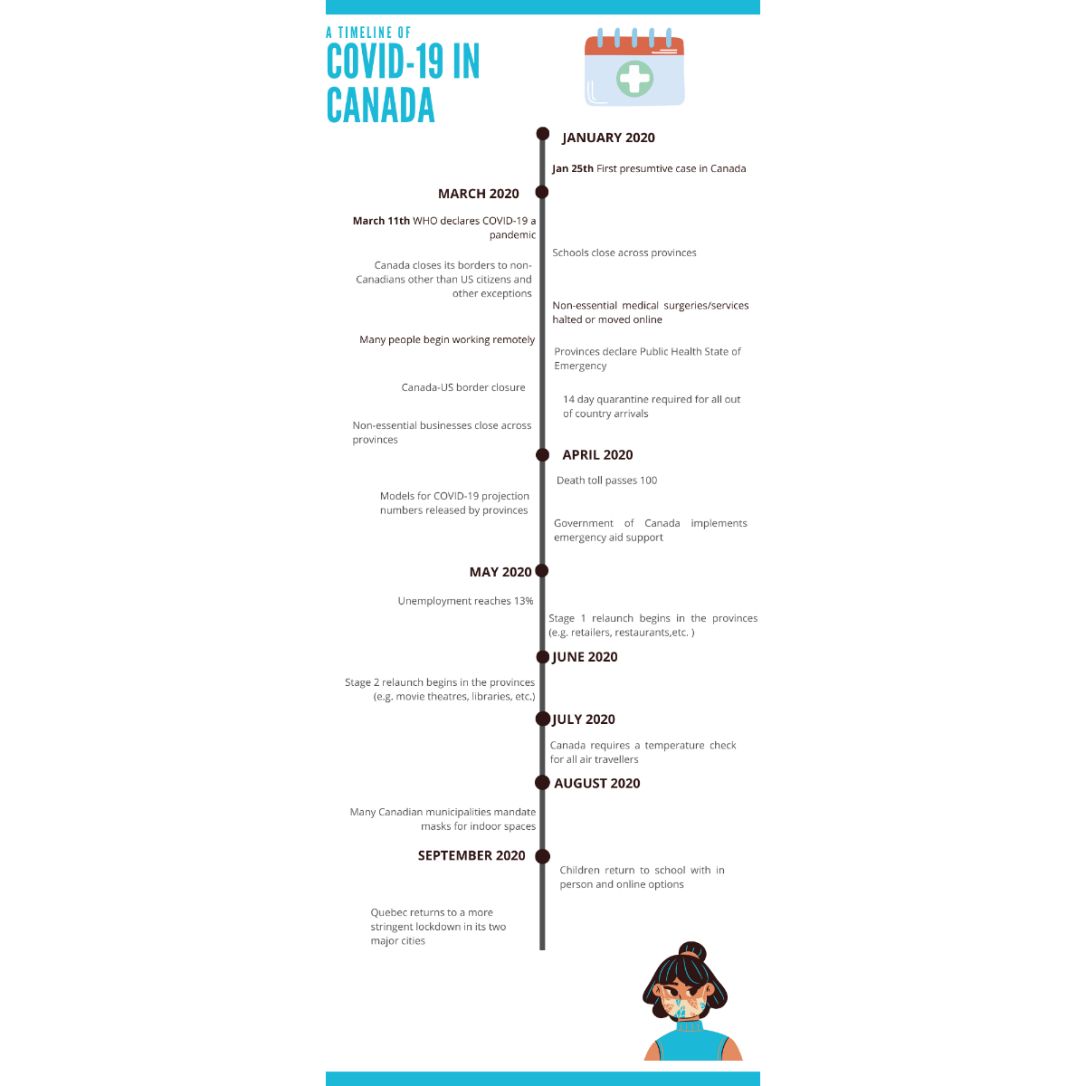
Timeline of COVID-19 events.

In December 2019, the novel SARS-CoV-2 caused an outbreak of COVID-19 in Wuhan, China, which rapidly spread around the world. COVID-19 was declared a global pandemic on March 11, 2020 [1]. Although most people with COVID-19 recover from the disease, the associated morbidity and mortality, as well as the uncertainty surrounding long-term effects, has prompted governments around the world to implement public health measures to slow and reduce the spread of COVID-19. Worldwide, these measures have included recommendations for hand and respiratory hygiene (ie, frequent handwashing; avoiding touching one’s eyes, nose, and mouth; coughing or sneezing into a bent elbow), travel restrictions, self-isolation, wearing masks in public, and physical distancing. These measures resulted in dramatic changes in the everyday life for most people, including the ways that people work, socialize, eat, and play. For example, many people were mandated to work from home, and a large number of people lost their jobs (both temporary and permanent) or saw substantial changes in their jobs. Schools and daycare centers were closed, with controversial reopening plans. Hospitals, health care facilities, and care homes limited their services and restricted visitor, caregiver, and support person access to patients and residents. Recreational facilities were closed, and public health recommendations to remain at home for anything other than essentials severely restricted people’s opportunities for recreation, physical activity, and socializing. These examples illustrate the disruption across many aspects of everyday life and the potential for uncertainty, worry, and fear that result from public health measures to limit the spread of COVID-19. A timeline of the major public health measures implemented in Canada is illustrated in Figure 1.

### A Need to Understand the Effects of Pandemics on Mental Health

Although there is evidence that *disasters* increase symptoms and incidence of mental illness, research on the mental health consequences of epidemics and pandemics is sparse. Existing studies focus on front-line health care workers [2] and the mental health consequences of adjusting to morbidity caused by disease (eg, parents of children with congenital zika virus syndrome) [3]. However, little is known about the psychological effects of pandemic countermeasures such as quarantine, physical distancing, and shelter-in-place orders. A small study of individuals instructed to voluntarily quarantine during the 2003 severe acute respiratory syndrome (SARS) outbreak in Toronto reported elevated symptoms of posttraumatic stress disorder and depression [4]. Postdisaster studies have consistently observed increases in mental health problems following large-scale but localized events such as natural (eg, earthquakes) [5], traumatic (eg, the World Trade Center attacks) [6], and environmental (eg, Chernobyl nuclear disaster) [7] disasters.

Although most individuals will display resilience in the face of disaster, a substantial proportion will experience some psychological impairment and a smaller proportion will develop a mental health disorder [8]. The degree or severity of exposure to disaster consistently and strongly predicts greater postdisaster psychological impairment [9], with additional contributions from factors such as predisaster mental health problems, low socioeconomic status, minority ethnicity, low social support, younger age, caring for children, and personality characteristics such as neuroticism [8]. A national population-based cohort study conducted in the United Kingdom found that minority ethnicity, overweight or obesity, age over 35 years, and pre-existing health conditions increased the risk for severe COVID-19 (eg, hospitalization required) among pregnant women [10].

There is growing concern that the public health response to the COVID-19 pandemic has created a shadow pandemic of mental illness. Public polls have repeatedly suggested widespread and dramatically elevated worries related to financial, social, and psychological well-being [11]. For example, 37% of the respondents of a nationally representative poll in the United States conducted on April 25-27, 2020, reported feeling overwhelmed from trying to work at home and balance other needs of their family [12]. A review of studies on anxiety or depression during the COVID-19 pandemic reported a prevalence of 26% and 31%, respectively [13]. Other studies have suggested that young people and women appear to be disproportionally affected by the COVID-19 pandemic [14-16].

Pregnant individuals may be particularly vulnerable to the negative psychological effects of public health measures related to COVID-19 [17], both because they represent the demographic most affected by disasters and because pregnancy itself entails significant life changes that require major psychosocial and emotional adjustments [18]. An early report from the current cohort [19] and other cohorts around the world [20-26] show that symptoms of depression and anxiety have increased dramatically among pregnant individuals during the COVID-19 pandemic, with greater fears surrounding social isolation and disease appearing to predict a greater risk of elevated symptoms.

Physical distancing policies implemented as a countermeasure to the spread of COVID-19 are especially concerning because social support buffers the negative effects of prenatal distress on both the mother and her offspring [27,28]. Social support and community cohesion are primary protective factors in the face of large-scale stressful events [29-31], and these factors may also apply to pandemics because countermeasures are known to increase a sense of social isolation [4].

### Effects of Prenatal Psychological Distress on Birth Outcomes and Child Development

The prenatal period is a time of vulnerability for the fetus during which maternal psychological distress can have deleterious effects on fetal development. Sustained prenatal psychological distress increases the risk of prenatal and postpartum depression, prenatal infection and illness [32], miscarriage, preterm birth, and reduced birthweight [33-37]. Furthermore, children that are prenatally exposed to maternal psychological distress are more likely to have physical, behavioral, cognitive, and emotional problems than their nonexposed peers, and they are at higher risk for physical and mental health problems at a later stage [33,38-42]. Specifically, regarding disasters, a series of reports on children born to mothers exposed to the 1998 Quebec ice storm found reduced cognitive and linguistic ability [43], increased risk for obesity [44], broad changes in DNA methylation [45], and increased amygdala volume, which mediated the association between prenatal maternal stress and higher levels of externalizing behavior in these children [46]. Together, findings from disaster studies suggest that increases in psychological distress following stressful events constitute a major public health concern for physical and mental development in the generation of children prenatally exposed to the current COVID-19 pandemic.

There have also been some unexpected aspects of the COVID-19 pandemic in the context of infants. Several studies during the early pandemic [47,48], although not all [49,50], reported substantially increased rates of stillbirth and reduced rates of preterm birth and very low birthweight. The decrease in the rates of preterm births appears to be temporary, with rates increasing to their more usual levels as the pandemic wears on [51]. Pregnancy cohorts would serve an important role in identifying factors contributing to any changes in birth outcomes with significant potential to improve child development outcomes if the lessons learned can be applied to postpandemic obstetric care.

It is important to note that developmental outcomes of offspring prenatally exposed to maternal psychological distress are heterogeneous, and this heterogeneity strongly suggests that risk and resilience factors operate to increase or decrease the effects of prenatal exposures on maternal and offspring outcomes [52]. Emerging evidence during the current pandemic supports the notion that risk and resilience factors such as poverty, being a racial minority, psychological resources, and social support modulate the risk of contracting COVID-19 and for more severe psychological impairment during the pandemic [53-55]. Risk and resilience factors are likely to also operate in relation to health outcomes for children born during the pandemic. It is therefore essential that risk and resilience factors for child outcomes can be identified early to optimally direct efforts to enhance resilience and reduce risk [56].

The COVID-19 pandemic presents a novel and unprecedented opportunity to study stress and resilience in humans not only because of its worldwide scope but also because people around the globe are faced with similar hardships that result from public health countermeasures (eg, job loss, social isolation, and disrupted access to health care). The current situation replicates important features of well-established paradigms to study stress susceptibility and resilience in animal models [57], where animals exposed to the same stressor or hardship nevertheless show dramatically different behavioral, immunological, epigenetic, and neurobiological responses [58-60]. Stress-susceptible individuals exhibit considerable changes in behavioral and neurobiological responses, whereas stress-resilient individuals exhibit small or temporary changes in behavior and neurobiology. We propose that objective exposure to hardships caused by the pandemic and pandemic countermeasures constitute a major prenatal stressor and that outcomes in children will differ as a function of maternal susceptibility or resilience. Specifically, we postulate that, in general, greater objective exposure to prenatal hardship because of the pandemic will be associated with poorer maternal and infant outcomes. We also expect this effect will be moderated by maternal psychological response such that low or temporary increases in maternal distress (ie, stress resilience) will be associated with less severe outcomes compared to outcomes among mothers with similar exposure to objective hardship but with large increases in maternal distress (ie, stress susceptible).

In addition to risk and resilience factors, fetal sex and timing of exposure during gestation make significant contributions to infant outcomes. Although different effects for boys and girls and across pregnancy trimesters are commonly reported, the overall findings are heterogeneous and difficult to summarize. The most pronounced sex differences have been observed for child neural or nervous system development and temperament outcomes [61]. Timing effects likely reflect vulnerabilities to environmental input at specific points in gestation (ie, sensitive periods), suggesting that it is not possible to define *the specific time* at which stress exposures have the greatest effects but rather that timing effects can only be specified in terms of specific outcomes [62]. For example, several large studies on stress exposure during pregnancy found the strongest associations with poor *birth outcomes* and *behavioral disorders* when exposure occurred in the second trimester [63-65], but the strongest association with *affective disorders* was reported when exposure occurred in the first trimester [66], and the strongest associations with *neurodevelopmental disorders* were reported in the third trimester [67]. However, some exposures can have opposite effects on the same outcome depending on gestational timing; for example, lower cortisol levels in early pregnancy but higher cortisol levels in later pregnancy are associated with more optimal cognitive outcomes in infants [68]. Taken together, these findings indicate the importance of including sex and exposure timing when considering the effects of the COVID-19 pandemic on offspring outcomes.

### Study Purpose

The Pregnancy During the COVID-19 Pandemic (PdP) study was designed to investigate the associations among exposure to objective hardship caused by the pandemic, perceived stress and psychological distress in pregnant individuals, and developmental outcomes in their offspring. The findings of this study are intended to provide knowledge about the psychological consequences of pandemics on pregnant individuals and their offspring and point toward prevention and intervention targets.

## Methods

### Primary Aims and Hypotheses

#### Aim 1

This study aims to determine the associations among objective exposure to hardship, perceived stress, and psychological distress among pregnant individuals during the COVID-19 pandemic. We hypothesize that greater exposure to COVID-19 stressors (eg, job loss or financial strain, death of a family member) will be associated with increased symptoms of depression, anxiety, and subjective stress.

#### Aim 2

This study aims to determine whether prepandemic risk factors increase vulnerability to objective COVID-19 hardship. We hypothesize that drug and alcohol use prior to pregnancy, adverse childhood experiences, minority ethnicity, low educational attainment, and poverty will increase the association between objective COVID-19 hardship and maternal psychological distress.

#### Aim 3

This study aims to determine whether resilience factors decrease psychological distress among pregnant individuals during the COVID-19 pandemic. We hypothesize that higher partner support, better sleep quality, and more physical activity will moderate or buffer associations between objective COVID-19 hardship and maternal psychological distress.

#### Aim 4

This study aims to determine the associations among exposure to prenatal objective hardship, perceived stress and psychological distress, and child development outcomes. We hypothesize that objective exposure to COVID-19 hardship will be more strongly associated with poor child development among stress-susceptible individuals (who also show high levels of subjective stress and psychological distress) than among stress-resilient individuals (who have low levels of subjective stress and psychological distress despite high exposure to objective hardship).

### Secondary Aims (Hypothesis Generation)

#### Aim 5

This study aims to determine whether gestational age at the onset of the COVID-19 pandemic is associated with infant outcomes. It is likely that each child outcome will be more strongly associated with exposure onset at some timepoints than at other timepoints during pregnancy.

#### Aim 6

This study aims to identify unique features of the COVID-19 pandemic that are particularly associated with increased psychological distress. We will explore potential changes in diet, physical activity, abuse, social connection, caregiving, employment, and finances.

#### Aim 7

This study aims to identify unexpected aspects of the pandemic that may contribute to mental wellness or distress. We will explore potential changes in family closeness, reduction in preterm birth, and positive aspects of working from home.

### Study Design and Procedures

The PdP study comprises a prospective longitudinal cohort of pregnant individuals (at enrollment) with repeated follow-ups during pregnancy and the postpartum period. Study enrollment, consent, and administration of questionnaires were conducted through Research Electronic Data Capture (REDCap) [69]. Advertisements through social media (Facebook and Instagram) directed potential participants to the study website [70] where they completed the eligibility survey and enrolled into the study. All participants signed the electronic consent form before proceeding to the first questionnaire.

**Figure 2:**
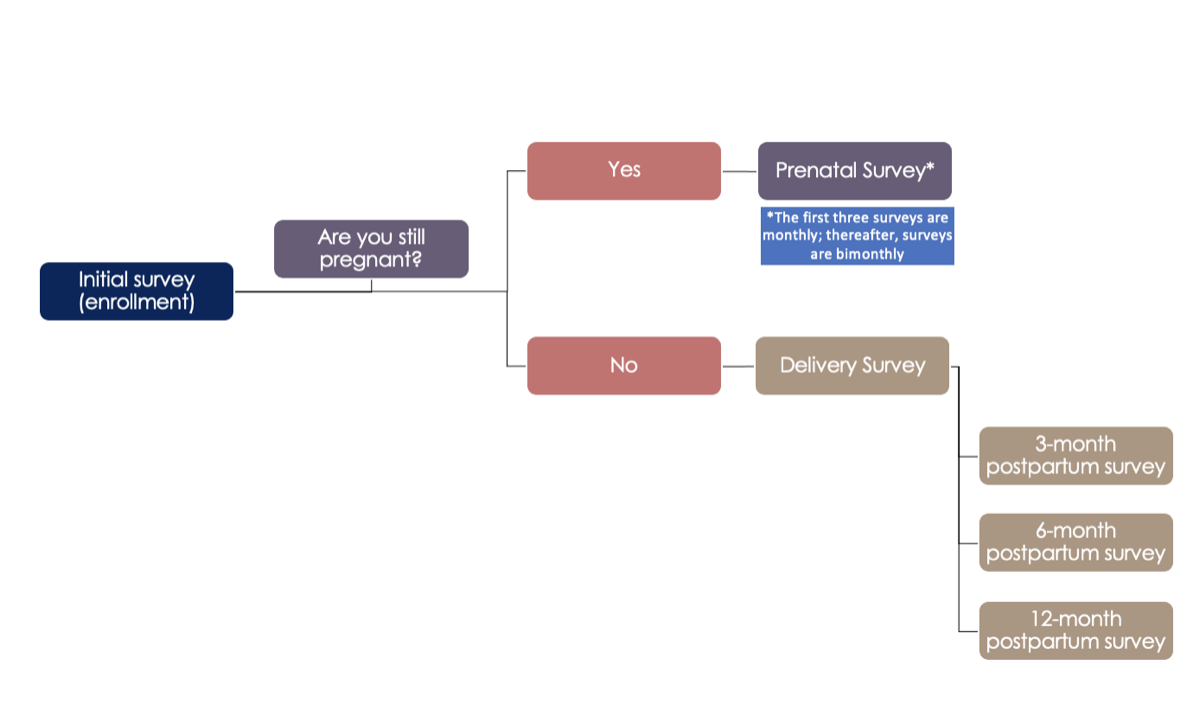
Study flow diagram.

An overview of the study procedure is presented in Figure 2. At enrollment, participants completed the initial survey that assessed demographic, socioeconomic, and obstetric characteristics, including age, postal code, ethnicity, household income, employment, marital status, education, country of origin, food insecurity, housing stability, history of previous pregnancies and births, prepregnancy health, prepregnancy height and weight, current weight, health conditions prior to and during pregnancy, medications, and other measures listed below. For the first three months following the initial survey, participants received a monthly email link to complete a follow-up survey that asked about their experiences since the previous survey. After three months, the follow-up surveys were sent every other month to reduce participant burden. Thus, participants completed a maximum of five prenatal follow-up surveys in addition to the initial survey. For each of these surveys, participants were first asked if they were still pregnant, and their answer to this question routed them either to the next prenatal survey or to the delivery survey. In the postpartum period, surveys were sent at 3, 6, and 12 months of infant age. A complete list of measures and timing of data collection are presented in Table 1.

**Table 1:** Study measures and timepoints.

Study measures		Timepoints								
		Prenatal period						Postnatal period		
		Initial survey	Prenatal follow-up (1-5)	Delivery			3 months		6 months	12 months
**General measures**										
	Eligibility questions and consent	✓								
	Demographic information	✓								
	Health behavior (eg, substance use, physical activity, and diet)	✓	✓				✓		✓	✓
	Prior, current, and changes in medical history and conditions	✓	✓				✓		✓	✓
	Pandemic Objective Hardship Scale	✓	✓				✓		✓	✓
	Perceived COVID-19 threat	✓	✓				✓		✓	✓
	Distress thermometer	✓	✓						✓	✓
	Edinburgh Postpartum Depression Scale (EPDS)	✓	✓				✓		✓	✓
	Pregnancy-Related Anxiety Questionnaire (PRAQ/PRAQ-R2)	✓	✓							
	PROMIS^a^ Anxiety	✓	✓				✓		✓	✓
	PROMIS Anger	✓					✓			✓
	PROMIS Sleep-Related Impairment	✓	✓							
	PROMIS Sleep Disturbance	✓	✓							
	Perceived Stress Scale (PSS)		✓	✓						
	Connor-Davidson Resilience Scale (CD-RISC-2)	✓	✓							
	Social isolation	✓	✓				✓		✓	✓
	Couple’s Satisfaction Index (CSI)	✓	✓				✓			✓
	Social Support Effectiveness Questionnaire (SSEQ)	✓							✓	
	Interpersonal Support Evaluation List (ISEL)	✓								
	Intolerance of Uncertainty Scale- Short form (IUS)	✓								
	Physical abuse (PRAMS^b^)	✓	✓						✓	✓
	Adverse Childhood Experiences (ACEs)^c^		✓	✓			✓			
	The Everyday Discrimination Scale (EDS)^c^		✓	✓						
	Self-compassion^c^		✓	✓						
	Intended infant feeding		✓	✓						
	Gender identity and sexual orientation questions^c^		✓	✓						
**Delivery measures**										
	Delivery type and outcome (live birth, miscarriage, and neonatal death)			✓						
	Baby information and health (weight, length, sex, and NICU^d^ stay)			✓						
	Birth experience and COVID-19 restrictions during birth or NICU stay			✓						
	Initial breastfeeding questions			✓						
	COVID-19 impact on breastfeeding and bonding			✓						
**Child development measures**										
	Infant health								✓	✓
	Ages and Stages Questionnaire, Third Edition (ASQ-3)									✓
	Ages and Stages Questionnaire: Social-Emotional, Second Edition (ASQ:SE-2)									✓
	Brief Infant Sleep Questionnaire (BISQ)						✓			✓
	The Infant Behaviour Questionnaire–Revised Very Short Form (IBQ-R)								✓	✓
	Crying patterns						✓			
	Infant feeding						✓		✓	✓
	COVID-19 disruptions to mothers’ postpartum services						✓		✓	✓
	COVID-19 disruptions to infant appointments and services						✓		✓	✓

^a^PROMIS: Patient-Reported Outcomes Measurement Information System.

^b^PRAMS: Pregnancy Risk Assessment Monitoring System.

^c^These measures were only collected once.

^d^NICU: neonatal intensive care unit.

### Study Population

Participants included individuals who were pregnant during the COVID-19 pandemic. Participants were considered eligible if they were ≥17 years, ≤35 weeks of gestation at the time of enrollment, living in Canada, and able to read and write in English or French. There were no additional exclusion criteria. The requirement of ≤35 weeks of gestation at study enrollment was intended to allow us to collect multiple data points during a participant’s pregnancy. However, we note that, based on their due dates, some participants were at >35 weeks of gestation at initial enrollment but otherwise provided legitimate data. We plan to retain these participants for potential secondary analyses, as relevant.

**Figure 3:**
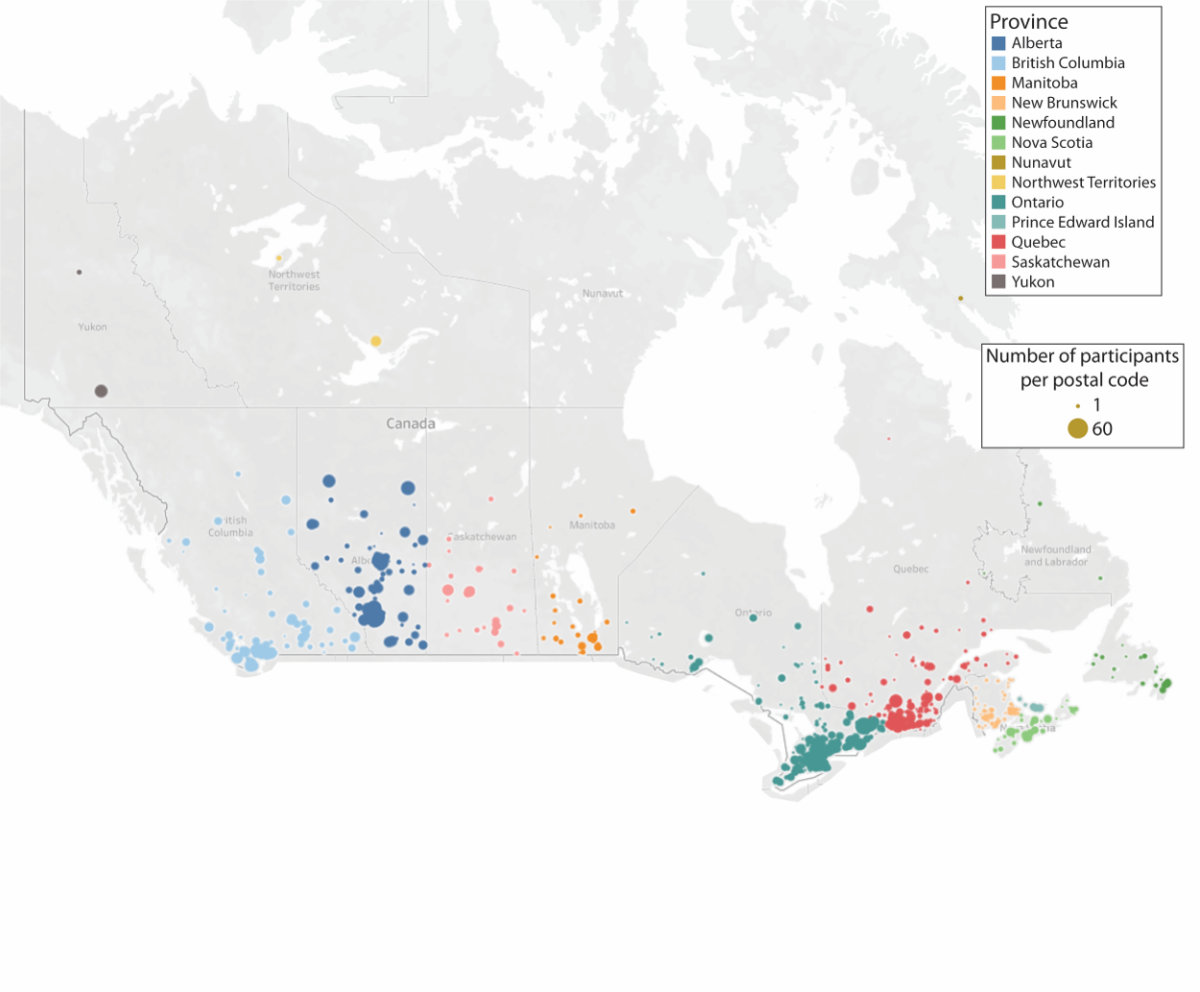
Geographic distribution of study participants by postal code. Figure generated using Tableau Maps.

Participant recruitment began on April 5, 2020, and it is currently ongoing. The recruitment goal is to obtain 9200 completed baseline surveys (see *Sample Size Considerations* below). To ensure broad representation, our advertising and surveys were available in both official Canadian languages (ie, French and English), and our social media ads target geographic regions and/or sociodemographic groups with less representation in the cohort (eg, rural communities in Northern Canada). Figure 3 provides a graphical summary of the geographic distribution of participants across Canada enrolled in this study to date.

### Study Measures

#### Maternal Measures

##### Depression Symptoms

Maternal depression symptoms experienced by the participants in the past week were assessed using the Edinburgh Postpartum Depression Scale (EPDS) [71], a self-report questionnaire with possible scores ranging from 0 to 30, where higher scores indicate more severe symptoms. A cut-off score ≥13 is used to identify individuals with clinically concerning depression symptoms [71]. Scores ≥13 during pregnancy have a sensitivity and specificity of 100% and 87%, respectively, for classifying major depression, and a positive predictive value of 33 [72]. This scale has been validated for both prenatal and postnatal assessment [73,74].

##### Anxiety Symptoms

General anxiety symptoms experienced by the participants in the past week were assessed using the 7-item Patient-Reported Outcomes Measurement Information System (PROMIS) Anxiety–Adult Short Form [75]. We follow the standard practice of converting raw scores to T-scores using the US general population norms; possible scores range from 36.3 to 82.7, with a mean score of 50 (SD 10). T-scores in the range of 60-69.9 are indicative of moderately elevated anxiety symptoms, and scores ≥70 are considered indicative of severely elevated anxiety symptoms [76].

Pregnancy-related anxiety symptoms (ie, worries about the health of the baby, birth, and caring for a new baby) experienced by the participants were assessed using the 10-item Pregnancy Related Anxiety Questionnaire (PRAQ) [77] in the English-language survey and the French translation of the 10-item Pregnancy Related Anxiety Questionnaire–Revised 2 (PRAQ-R2) in the French-language survey [78]. Possible scores on the PRAQ range from 10 to 40 and those on the PRAQ-R2 range from 10 to 50. Both questionnaires have acceptable internal consistency (Cronbach α=.80-.81) [78,79], and their validity is supported by extensive use in relation to both maternal and infant outcomes. There are no cut-off scores for these scales, but previous treatment studies have used a median split to define groups with higher versus lower pregnancy-related anxiety symptoms [80], with higher scores indicating more severe symptoms.

##### Anger

Anger experienced by the participants in the past week was assessed using the 5-item PROMIS Short Form v1.1–Anger 5a [75]. As with other PROMIS measures, raw scores are converted to T-scores. T-scores range from 32.9 to 82.9; the mean score was 50 (SD 10), with scores in the range of 60-69.9 indicative of moderately elevated anger and scores ≥70 indicative of severely elevated anger [76].

##### Distress Thermometer

The Distress Thermometer was used to measure the overall level of subjective distress experienced by the participants in the previous week, on a visual analog scale of 0 (“Not distressed”) to 10 (“Extremely distressed”) [81]. A cut-off score ≥4 is typically used to signify clinically concerning distress [82]. The Distress Thermometer has demonstrated good validity and temporal stability [83,84].

##### Perceived Psychological Stress

Participants’ subjective experience of psychological stress over the past month was assessed with the widely used 10-item Perceived Stress Scale (PSS) [85]. The PSS measures the degree to which participants appraise their lives as unpredictable, uncontrollable, and overloaded [86]. Scores range from 0 to 40, with higher scores indicating greater perceived stress. Reliability and validity of the PSS have been supported across multiple studies [85].

##### Pandemic Objective Hardship Scale

COVID-19 represents a novel exposure for which there are no previously developed measures. Nevertheless, previous work on objective measures of hardship resulting from exposure to natural disasters provided a principled and systematic framework for developing a new measure [87]. For instance, work by King and Laplante [87] has identified four major components of disaster exposures, which were adapted to the COVID-19 context: scope, loss, threat, and change. Scope refers to the duration and intensity of the hardship, with the former referring to the amount of time for which major aspects of participants’ lives were disrupted and the latter focusing on the number of individuals within participants’ communities who were similarly affected. Loss refers to financial, social, and physical losses experienced as a result of the pandemic. For example, the loss of employment, savings, closures of schools, and daycares represent relevant losses. Threat refers to physical and health-related consequences of exposure to the stressor. For example, being infected with SARS-CoV-2 or hospitalization of a close friend with COVID-19 represent threats to self and others. Change captures the adjustments to daily living, prenatal care, work, and social interaction caused by the COVID-19 pandemic. Relevant changes include working from home, altering a birth plan, and reductions in physical activity or diet quality. The timeframe for these items was the previous month or the previous questionnaire (ie, up to three months).

##### Prenatal and Postnatal Care

Changes and disruptions to prenatal and postnatal care received by the participants were assessed using a series of questions tailored to our study relating to the way that prenatal care was delivered, cancellations, ability to bring partner to appointments, and changes to birth plans. Participants are also asked to evaluate the impact of these changes on the quality of care they and their baby have received in the past three months.

##### Perceived COVID-19 Threat

The degree to which participants feel that COVID-19 was a threat to their health or the health of their baby at any time during the pandemic was assessed by three items developed for the study: (1) “How much do (did) you think your life is (was) in danger during the COVID-19 pandemic?” (2) “How much do (did) you think your baby’s life is (was) in danger at any time during the COVID-19 pandemic?” and (3) “How much are you worried that exposure to the COVID-19 virus will harm your baby?” Responses were scored on a 100-point sliding scale, with the left anchor indicating 0 points (“Not at all”); the middle anchor, 50 points (“Somewhat”); and the right anchor, 100 points (“Very much so”).

##### Adverse Childhood Experiences

Adverse childhood experiences (ACEs) of the participants were assessed using a 10-item measure [88] of early-life adversity (age 0-18 years) across three domains: abuse (emotional, physical, and sexual), neglect (physical and emotional), and exposure to household dysfunction (domestic violence, substance abuse, mental illness, parental separation or divorce, and incarcerated household member). The ACEs questionnaire is widely used and has demonstrated good reliability and internal consistency (Cronbach α=.81) [89], as well as adequate test-retest reliability (weighted κ=0.64) [90]. The occurrence of individual ACEs is summed to create the ACE score with a potential range of 0-10. Based on previous work that has examined *dose-response* relationships, participants were coded as having experienced 0, 1, 2, 3, or ≥4 ACEs [88,91,92].

##### Perceived Social Support

Participants’ perception of the quality of social support received from their romantic partner over the previous three months was assessed using the 35-item Social Support Effectiveness Questionnaire (SSEQ) [79]. Within the domains of emotional, informational, and task support, participants were asked to rate their experience over the past three months on a 5-point scale: (1) how well the quantity of support received from their partner matched the amount they wanted, (2) whether they wished the support had been different somehow, (3) how skillful their partner was at providing support, (4) how often it was difficult to solicit support, and (5) whether their partner offered support without being asked. Participants also rated the extent to which they perceived their partner’s support as negatively infringing upon their self-efficacy or self-esteem. Internal consistency of the SSEQ is strong (Cronbach α=.87), and it has previously been used to distinguish levels of social support in samples of pregnant individuals [79,93,94]. Total scores can range from 0 to 80, with higher scores indicating more effective support.

Perceived social support was also assessed using the 12-item Interpersonal Support Evaluation List (ISEL) [95,96], to determine appraisal (eg, advice or problem solving), belongingness (eg, shared experiences), and tangible support (eg, help with daily chores) over the past three months. Here, we focus on the total score, which is the sum of the three subscales. Scores range from 12 to 48, with a higher score indicating greater perceived support.

##### Social Isolation

Feelings of social isolation were assessed using the following item: “During the COVID-19 pandemic, I have felt more alone than usual.” Participants’ responses were on a 100-point sliding scale, with the left anchor indicating 0 points (“Not at all”); the middle anchor, 50 points (“Somewhat”); and the right anchor, 100 points (“Very much so”).

##### Relationship Quality

Participants’ perception of partner relationship quality was assessed using the 4-item Couple Satisfaction Index (CSI-4) [97]. Scores range from 0 to 21, with higher scores indicating higher levels of relationship satisfaction. CSI-4 scores below 13.5 suggest notable relationship dissatisfaction. Changes in relationship quality as a function of the pandemic were assessed for relationships with partner, children, and close friends and family by using items generated for the study. For each relationship type, participants were asked how the COVID-19 pandemic affected their relationship. Responses were recorded on a 100-point sliding scale, with the left anchor indicating 0 points (“It has strained our relationship”); the middle anchor, 50 points (“Not much has changed”); and the right anchor, 100 points (“It has brought us closer together’”).

##### Physical Abuse

Experiences of physical harm in the 12 months prior to pregnancy and during the current pregnancy were assessed using two items adapted from the Pregnancy Risk Assessment Monitoring System (PRAMS) [98]. These items were queried with regard to the 12-month periods before pregnancy, since the last survey, and after giving birth.

##### Physical Activity

Participants reported their physical activity using a modified form of the Godin-Shephard Leisure-Time Exercise Questionnaire (GLTEQ) in a typical week over the past month [99]. Participants reported the number of days per week in which they engaged in mild (eg, light walking), moderate (eg, brisk walking), and strenuous (eg, running) exercise of more than 15 minutes. Definitions of each category of physical activity were provided. The total score was calculated per standard scoring procedure for GLTEQ, by multiplying episodes of mild exercise by 3, moderate exercise by 5, and strenuous exercise by 9. Participants with scores below 14 are considered sedentary, those with scores ranging from 14 to 23 are considered moderately active, and those with scores equal to or more than 24 are considered active. An additional item was included asking participants how their level of physical activity changed because of the COVID-19 pandemic. Responses were recorded on a 5-point Likert-type scale with the following options: “Substantially decreased” (1 point), “Somewhat decreased” (2 points), “No change” (3 points), “Somewhat increased” (4 points), and “Substantially increased” (5 points).

##### Sleep Quality

Sleep disturbance and impairment in the past week were assessed using the 4-item PROMIS Sleep Disturbance–Short Form 4a and the 4-item PROMIS Sleep-Related Impairment–Short Form 4a [100]. As with other PROMIS measures, raw scores are converted to T-scores. T-scores ranging from 60 to 69.9 are considered moderate problems, and scores ≥70 are considered severely elevated. Sleep duration (ie, hours of sleep per night) was assessed with a single item from the Pittsburgh Sleep Quality Index [101].

##### Diet

Changes in diet and eating patterns were assessed using a questionnaire developed for the study to determine how participants’ diet during the COVID-19 pandemic differed from their prepandemic diet and the reasons for the change. Dietary changes were assessed in the following categories: fresh vegetables or fruits, dairy, meats, canned or dried foods, *fast food*s, take-out or home delivery, and sweets or snacks. For each category, participants responded on a 5-point Likert-type scale, with the following options: “I eat much more” (1 point), “I eat more” (2 points), “I eat about the same” (3 points), “I eat less” (4 points), and “I eat much less” (5 points). If participants reported a change in their diet (ie, did not select “I eat about the same” option), then they were asked about the reason for the change with the following response options: “Can no longer afford,” “Can’t go grocery shopping frequently,” “Can spend more time cooking and preparing food,” “Change in craving,” and “Other (specify below)” (scores: Yes=1, No=0). Although changes in craving were not expected as a direct result of the pandemic, they are commonly reported during pregnancy [102]; it was therefore important to disambiguate such pregnancy-related changes in diet from changes that are more directly related to the pandemic.

##### Substance Use

Substance use prior to (ie, in the 12 months before pregnancy) and during the current pregnancy (ie, in the past month) were assessed using a self-report measure separately for alcohol, cannabis, tobacco, and illicit drugs. Participants were asked how many days per week they consumed each substance and how many drinks or products per day they typically consumed. Participants were also asked whether they changed their use patterns in pregnancy and if they had, they were asked when they changed their use patterns.

##### Coping and Resilience

Participants’ perceived ability to cope with stressful situations was assessed with the 2-item Connor-Davidson Resilience Scale (CD-RISC 2), which demonstrates good test-retest reliability and convergent and divergent validity [103]. Each item is rated on a 5-point Likert scale ranging from 0 (“Not true at all”) to 4 (“True all the time”); total scores can range from 0 to 8, with higher scores indicating more successful coping. We also included an open-ended question about coping: “People are responding to the pandemic in many ways. Can you tell us what things you are doing to cope with the COVID-19 pandemic?”

The 27-item Intolerance of Uncertainty Scale (IUS) was used to measure the extent to which participants believe uncertainty is stressful, upsetting, negative, unfair, and leads to the inability to take action [104,105]. Items are rated on a scale ranging from 1 (“Not at all characteristic of me”) to 5 (“Entirely characteristic of me”). Previous research shows that the IUS has excellent internal consistency (Cronbach α=.91) and good test-retest reliability (*r*=0.74), and it is highly correlated with symptoms of generalized anxiety disorder [104].

Self-compassion, a trait level form of resilience, was assessed using the 12-item short form of the Self-Compassion Scale–Short Form (SCS-SF) [106]. The SCS measures the tendency to treat oneself with kindness and understanding rather than harsh self-judgment, to recognize imperfections and suffering as part of the human experience, and to have mindful awareness and tolerance of negative thoughts and feelings. Each item is rated on a 5-point Likert scale ranging from 1 (“Almost never”) to 5 (“Almost always”); total scores range from 10 to 60, with higher scores indicating more self-compassion. The SCS has adequate internal consistency (Cronbach α=.86) and strong correlation (*r*=0.97) with the long form [106].

##### Discrimination

Participants’ experiences of discrimination were assessed using the 5-item Everyday Discrimination Scale [107]. This questionnaire asks about day-to-day experiences of discrimination, including being treated differently than other people and feeling threatened or harassed. Responses items ranged from 0 (“Never”) to 6 (“Almost every day”). For items rated as more than “A few times a year,” participants were also asked a follow-up question about what they think is the main reason for these experiences.

##### Vaccination

Participants were asked if they planned to receive a COVID-19 vaccine, if they had received a COVID-19 vaccine (which vaccine, if yes), and the dates of the doses (or the gestational age if they were still pregnant).

#### Child Development Measures

Parent report of child outcomes were assessed at 3, 6, and 12 months postpartum.

##### Developmental Milestones

The Ages and Stages Questionnaire, Third Edition (ASQ-3) [108] is a widely used parent-reported and norm-referenced developmental screening tool [109] that assesses delays in child development across five domains: communication, gross motor, fine motor, problem-solving, and personal adaptive skills. The ASQ-3 has been identified by the American Academy of Pediatrics as a high-quality tool for use in clinical practice to screen for delayed developmental milestones in children [110]. Sensitivity and specificity of the ASQ-3 are both 86% for distinguishing between children at risk for developmental delay and children not at risk. Parents rate each of the 30 items on a scale ranging from “Yes” (10 points), “Sometimes” (5 points), or “Not yet” (0 point) based on the infant’s current ability.

##### Socioemotional Development

The Ages and Stages Questionnaire: Social-Emotional, Second Edition (ASQ:SE-2) [111] is a validated and widely used parent-report screening tool in 7 areas of socioemotional development: self-regulation, compliance, social communication, adaptive functioning, autonomy, affect, and interaction with people. A total score is calculated to index overall socioemotional problems. Parents rate each of the 27 items on a scale ranging from “Often or always” (0 point), “Sometimes” (5 points), or “Rarely or never” (10 points) based on the infant’s usual behavior.

##### Temperament

The Infant Behavior Questionnaire–Revised Very Short Form (IBQ-R) [112,113] is a 37-item parent-report measure of infant temperament. Three broad dimensions of temperament are assessed: negative emotionality, regulatory capacity/orienting, and positive affectivity/surgency. Parents report their observations of specific infant behaviors in the past week using a 7-point Likert scale ranging from “Never” to “Always.” Each item also had a “Does not apply” option. Scores on each dimension can range from 1 to 7, with higher scores reflecting stronger evidence of each dimension. The IBQ-R has strong psychometric properties and is widely used in the child development literature [114,115].

##### Crying

Periods of persistent infant crying (defined as half an hour or more during which the baby would not settle) in the past week were assessed using several items from the Crying Patterns Questionnaire [116]. The validity of the Crying Patterns Questionnaire, relative to a cry diary, has been supported [117].

##### Infant’s Sleep Quality

Parents’ report of their infant’s sleep quality in the past two weeks was assessed using the 19-item Brief Infant Sleep Questionnaire (BISQ)–Revised Short Form [118]. In addition to a total score, three subscale scores are calculated: infant sleep (5 items), parent perception (3 items), and parent sleep-related behaviors (11 items). The measure is scored using an age-based and norm-referenced system [119]. The total score and each subscale score are scaled from 0 to 100, with higher scores indicating better sleep quality, more positive perception of infant sleep, and parent behaviors that promote healthy and independent sleep. The BISQ is widely used, and its reliability and validity have been documented [120,121].

##### Feeding

Based on previously published questionnaires [122,123], we assessed onset, duration, and proportion of breastfeeding and formula feeding by using a series of maternal-report questions that conform to the World Health Organization’s breastfeeding categories: exclusive breastfeeding, predominant breastfeeding, mixed feeding, and bottle feeding [124].

### Sample Size Considerations

To power the study adequately for each of the hypotheses, we conducted a sample size calculation for Hypothesis 5, which will test the timing effects of prenatal exposure on child development outcomes. We choose this hypothesis because it requires the largest sample, and adequately powering this hypothesis will also adequately power the other hypotheses. We used G*Power (version 3.1) [125] to estimate the sample size required to test differences in the proportion of children not meeting developmental milestones. About 26% of children in Canada did not meet developmental milestones in one or more area of development prior to the COVID-19 pandemic [126]. To detect an increase in proportion of 0.09 not meeting developmental milestones (which is equal to the interprovincial variability in the proportion of children not meeting developmental milestones), assuming a power of 0.90 and α=.001, a sample size of 2184 is required. To conduct adequately powered analyses stratified by trimester, we require a sample of 6552 with infant milestone data. Allowing for a 10% attrition due to miscarriage and stillbirth and an additional 30% attrition due to other reasons after the initial survey, we plan to recruit a total sample of 9200.

### Data Analysis

Data visualization and screening will be conducted to determine what, if any, data manipulations are required. Regression-based analysis are planned to address study hypotheses. Logistic regression will be used with categorical outcomes, and multivariable regression will be used for continuous outcomes. Longitudinal analyses will be conducted using multilevel modeling. All analyses will include covariates deemed important to control for confounding and to increase the precision of the model. Planned subgroup analyses include grouping based on established cut-off scores for measures of anxiety and depression symptoms, analyses comparing individuals with confirmed COVID-19 to those who did not have COVID-19, and analyses by child sex for child outcomes. We also plan to examine time-related factors, including timing effects related to trimester of pregnancy when the pandemic was declared and the influence of pandemic phase (ie, how stress, distress, and fear change over time) on outcomes. Missing data will be assessed to determine what treatments are required. Given the risk of attrition bias in longitudinal studies, we will use a missing data strategy that yields unbiased estimates (eg, maximum likelihood and multiple imputation).

### Ethical Considerations

This study received ethics approval (REB20-0500) from the University of Calgary Conjoint Health Research Ethics Board on March 26, 2020. All participants were required to voluntarily agree to participate in this study and sign the electronic informed consent form prior to providing any data.

### Data Management and Availability

Study data were collected and managed using REDCap electronic data capture tools hosted at the University of Calgary and the University of Alberta [69]. REDCap is a secure, web-based application designed to support data capture for research studies, providing (1) an intuitive interface for validated data entry, (2) audit trails for tracking data manipulation and export procedures, (3) automated export procedures for seamless data downloads to common statistical packages, and (4) procedures for importing data from external sources.

Metadata will be included in the Canadian Research Advancement through Cohort Cataloguing and Harmonization (REACH) project [127].

Data are available upon reasonable request made to the corresponding author.

## Results

Participant recruitment via social media (Facebook and Instagram) began on April 5, 2020, and is ongoing. As of April 2021, more than 11,000 individuals started the initial survey. Follow-up data collection is ongoing.

## Discussion

The design and implementation of this study protocol were executed during the early phase of the pandemic with data collection starting on April 5, 2020. Because of the evolving nature of the pandemic, some of the questions specific to the pandemic required modification, and additional questions were added to reflect the emerging issues. For example, beginning in June 2020, when provincial governments began to implement phased approaches to relaunching the economy, we added questions about perceptions of these relaunch plans.

Strengths of this study include its prospective longitudinal design, implementation at an early phase of the pandemic, sample size, recruitment (and representation) from every province and territory in Canada, a measure of objective exposure to pandemic hardships, use of measures with strong psychometric properties, and measurement of many potential confounding variables. Limitations include reliance on self-report measures that are not diagnostic in nature, the potential to attract participants with higher levels of psychological distress, the potential for attrition bias because of differential loss to follow-up, and the use of a cohort design that limits causal interpretation.

This longitudinal investigation seeks to elucidate the associations between hardships caused by the COVID-19 pandemic, maternal psychological distress, child development, and risk and resilience factors that amplify or ameliorate these associations. The findings of this study are intended to provide knowledge about the psychological consequences of pandemics on pregnant individuals and point toward prevention and intervention targets.

## Abbreviations

ACEs: Adverse Childhood Experiences
ASQ-3: Ages and Stages Questionnaire, Third Edition
ASQ: SE-2: Ages and Stages Questionnaire: Social-Emotional, Second Edition
BISQ-R: Brief Infant Sleep Questionnaire–Revised
CD-RISC 2: Connor-Davidson Resilience Scale
CSI-4: 4-item Couple Satisfaction Index
EDS: Everyday Discrimination Scale
EPDS: Edinburgh Postnatal Depression Scale
GLTEQ: Godin-Shephard Leisure-Time Exercise Questionnaire
IBQ-R: Infant Behavior Questionnaire–Revised
ISEL: Interpersonal Support Evaluation List
IUS: Intolerance of Uncertainty Scale
PdP: Pregnancy During the COVID-19 Pandemic
PRAMS: Pregnancy Risk Assessment Monitoring System
PRAQ: Pregnancy Related Anxiety Questionnaire (English)
PRAQ-R2: Pregnancy Related Anxiety Questionnaire–Revised 2 (French)
PROMIS: Patient-Reported Outcomes Measurement Information System
PSS: Perceived Stress Scale
REDCap: Research Electronic Data Capture
SARS: severe acute respiratory syndrome
SCS-SF: Self-Compassion Scale–Short Form
SSEQ: Social Support Effectiveness Questionnaire
